# Supplementary material for: Screening and Optimization of Solid-State Fermentation for Esteya vermicola, an Entomopathogenic Fungus Against the Major Forest Pest Pine Wood Nematode
Source: Microorganisms. 2025 Feb 17;13(2):434. doi: 10.3390/microorganisms13020434 (PMC11858419; doi:10.3390/microorganisms13020434)
Supplement: Supplementary file 1 [file microorganisms-13-00434-s001.zip › microorganisms-3407460-supplementary.pdf]

Screening and Optimization of Solid-State Fermentation for Esteya vermicola, an Entomopathogenic Fungus Against the Major Forest Pest Pine Wood Nematode

Journal: Microorganisms

Lanwen Zhang[1], Yongxia Li[1,2,3]*, Xiaojian Wen[1,2], Xuan Wang[1,2], Wei Zhang[1,2], Dongzhen Li[1,2], Yuqian Feng[1,2], Zhenkai Liu[1,2,3], Xingyao Zhang[1,2]

[1]Key Laboratory of Forest Protection of National Forestry and Grassland Administration, Ecology and Nature Conservation Institute, Chinese Academy of Forestry, Beijing 100091, China

[2]Co-Innovation Center for Sustainable Forestry in Southern China, Nanjing Forestry University, Nanjing 210073, China

[3]Kunyushan Forest Ecosystem National Observation and Research Station, Yantai 264100, China

*Corresponding author

Email: liyongxiaxjs@163.com

Supplementary Information

Table S1. The factors and levels of orthogonal test $L_{25}(5^4)$

Level	Factor			
	A: Wheat bran	B: Corn flour	C: Soybean flour	D: Substrate-to-water ratio
1	5	0	0	1： 0.5
2	6	1	1	1： 0.6
3	7	2	2	1： 0.7
4	8	3	3	1： 0.8
5	9	4	4	1： 0.9

Table S2. The factors and levels of orthogonal test $L_9(3^3)$

Level	Factor		
	E: Glucose	F: YEF	G: $MgSO_4$
1	1	1	1
2	2	2	2
3	3	3	3

Table S3. Lunate conidia production of Fxy121 in different SSF medium

NO.	Factor				Lunate conidia production ($\times 10^8$ conidia/g)
	A Wheat bran	B Corn flour	C Soybean flour	D Substrate-to-water ratio	
1	5	0	0	1:0.5	0.93±0.21
2	5	1	4	1:0.9	2.04±0.36
3	5	2	3	1:0.8	3.76±0.52
4	5	3	2	1:0.7	4.96±0.64
5	5	4	1	1:0.6	3.98±0.56
6	6	0	2	1:0.8	3.14±0.34
7	6	1	1	1:0.7	7.13±0.52
8	6	2	0	1:0.6	5.89±0.32
9	6	3	4	1:0.5	3.11±0.15
10	6	4	3	1:0.9	5.07±0.58
11	7	0	4	1:0.6	4.09±0.67
12	7	1	3	1:0.5	5.04±0.36
13	7	2	2	1:0.9	6.82±0.47
14	7	3	1	1:0.8	7.63±0.53
15	7	4	0	1:0.7	6.48±0.47
16	8	0	1	1:0.9	4.56±0.55
17	8	1	0	1:0.8	5.02±0.28
18	8	2	4	1:0.7	6.35±0.46
19	8	3	3	1:0.6	6.59±0.47

20	8	4	2	1:0.5	4.79±0.42
21	9	0	3	1:0.7	5.36±0.40
22	9	1	2	1:0.6	6.54±0.28
23	9	2	1	1:0.5	4.18±0.35
24	9	3	0	1:0.9	4.28±0.36
25	9	4	4	1:0.8	5.57±0.54

Note: Data are presented as mean ± standard deviation.

Table S4. The lunate conidia production of Fxy121 in SSF under different carbon sources, nitrogen sources and inorganic salt combinations

No.	Factor			Lunate conidia production (× 10^8 conidia/g)
	E Glucose	F YEF	G $MgSO_4$	
1	1	1	1	13.89±0.57
2	1	2	3	11.73±0.76
3	1	3	2	12.21±0.32
4	2	1	2	12.76±0.43
5	2	2	1	11.17±0.54
6	2	3	3	10.43±0.38
7	3	1	3	9.24±0.64
8	3	2	2	8.55±0.28
9	3	3	1	9.06±0.27

Table S5. Experimental design of Box-Behnken

Run No	A:Temperature (℃)	B: Humidity (%RH)	C: Inoculation volume(%(v/w))	D: Fermentation time(d)	Lunate conidia production (× 10^8 conidia/g)
1	24	70	15	7	13.24
2	26	70	15	7	12.72
3	24	90	15	7	12.85
4	26	90	15	7	12.07
5	25	80	10	6	14.73
6	25	80	20	6	14.98
7	25	80	10	8	15.07
8	25	80	20	8	15.37
9	24	80	15	6	13.34
10	26	80	15	6	12.61
11	24	80	15	8	13.87
12	26	80	15	8	13.15
13	25	70	10	7	14.94
14	25	90	10	7	14.42
15	25	70	20	7	15.05
16	25	90	20	7	14.38
17	24	80	10	7	14.56
18	26	80	10	7	13.77

19	24	80	20	7	14.91
20	26	80	20	7	14.05
21	25	70	15	6	14.46
22	25	90	15	6	13.87
23	25	70	15	8	14.64
24	25	90	15	8	14.03
25	25	80	15	7	15.87
26	25	80	15	7	16.22
27	25	80	15	7	16.81
28	25	80	15	7	16.44
29	25	80	15	7	16.58
